# The Association Between Foxp3 Polymorphisms and Risk of Graves' Disease: A Systematic Review and Meta-Analysis of Observational Studies

**DOI:** 10.3389/fendo.2020.00392

**Published:** 2020-06-16

**Authors:** Han-ning Li, Xing-rui Li, Ya-ying Du, Zhi-fang Yang, Zheng-tao Lv

**Affiliations:** ^1^Department of Thyroid and Breast Surgery, Tongji Medical College, Tongji Hospital, Huazhong University of Science and Technology, Wuhan, China; ^2^Department of Orthopedics, Tongji Medical College, Tongji Hospital, Huazhong University of Science and Technology, Wuhan, China

**Keywords:** Graves' disease, autoimmune thyroid disease, polymorphism, Foxp3, meta-analysis

## Abstract

**Purpose:** This systematic review and meta-analysis was carried out with the aim of investigating the relationship between Foxp3 polymorphisms (rs3761547, r3761548, and rs3761549) and the risk of Graves' disease (GD).

**Methods:** Four online database including PubMed, EMBASE, ISI Web of Science, and CNKI were searched to identify observational studies that evaluated the association between Foxp3 polymorphisms and risk of GD. The strength of associations was indicated as odds ratio (OR) and corresponding 95% confidence interval (95%CI) under the allelic model. The Newcastle-Ottawa Scale was used to assess the methodological quality. Pre-specified subgroup analysis and sensitivity analysis were performed using RevMan 5.3 software. Publication bias was detected by Egger's and Begg's tests.

**Results:** Eight case control studies involving 3,104 GD patients and 3,599 healthy controls were included. The methodological quality of included studies was considered to be moderate to high. The results of our meta-analysis supported no association of rs3761547 and risk of GD in Asians (OR: 1.07, 95%CI 0.97, 1.19, *P* = 0.18). Evidence for rs3761547 and GD risk among Caucasians was still limited because only one study reported marginally increased risk of GD with the minor allele of rs3761547 (*P* = 0.04). The variant allele of both rs3761548 (OR: 1.31, 95%CI 1.04, 1.64; *P* = 0.02) and rs3761549 (OR: 1.30, 95%CI 1.03, 1.64; *P* = 0.03) was associated with increased risk of GD among Asians, but neither polymorphism turned out to be related with GD among Caucasians.

**Conclusion:** Rs3761548 and rs3761549 polymorphisms in Foxp3 were associated with risk of GD among Asians, possibly due to suppressed function of regulatory T cells and augmented autoimmune response. Their genetic effect among Caucasians remained to be confirmed by future large-scale and well-designed studies.

Regulatory T cells (Tregs) are essential to prevent autoimmunity and have been indicated to mediate immune tolerance in the development of Graves' disease (GD), which represents both the most common cause of hyperthyroidism and a typical example of antibody-mediated autoimmunity. The development and function of Treg cells is controlled by forkhead box P3 (Foxp3). Therefore, genetic variations in Foxp3 gene could be plausible candidate loci that contributed to the development of GD by means of changing the expression level of Foxp3. As a matter of fact, several association studies have been conducted to test this hypothesis but obtained inconsistent results. Hence, we performed a meta-analysis based on the currently available evidence to quantitatively analyze the association between Foxp3 polymorphisms and risk GD. We found that rs3761548 and rs3761549 polymorphisms in Foxp3 were associated with risk of GD among Asians, possibly due to suppressed function of regulatory T cells and augmented autoimmune response. However, their genetic effect among Caucasians cannot be determined as only limited number of studies has been conducted so far. Evidence from our study provided evidences concerning the involvement of the genetic alterations of Foxp3 caused by gene polymorphisms in Tregs-related pathogenesis of GD.

## Introduction

Graves' disease (GD) is the most common cause of hyperthyroidism, characterized by the production of autoantibodies against the thyroid cells, diffuse goiter, and abnormal thyroid hormone production, affecting around 2% of the women and 0.2% of the men (1). In particular, GD is caused by autoantibodies against the thyroid stimulating hormone receptor (TRAb) causing persistent hyperthyroidism. Other thyroid autoantibodies such as those against thyroid peroxidase (TPOAb) or thyroglobulin (TgAb) may or may not be present. The definite etiology of GD remains unclear, but solid evidence suggested that both genetic factors and environmental risk factors contributed substantially to its pathogenesis, with genetic factors playing a stronger role in this process (2, 3). In conjunction with exposure to environmental risk factors such as smoking, virus infection, iodine intake as well as use of certain drugs, genetically predisposed individuals are more likely to develop GD (2). So far, variations in several genes, in particular immune regulatory genes, have been found to be closely related with the development of GD, including CD40 (4), vitamin D receptor (5), PTPN22 (6), STAT4 (7), IL-6 (8), IL-2 (9), IL-2RA (10), IL-10 (11), TSHR (12, 13), TG (14), and CTLA4 (15). Similar mechanisms could also be implicated in the pathogenesis of Graves' ophthalmopathy (GO), which affects up to half of the patients with GD.

Regulatory T cells (Tregs) are a unique subpopulation of T cells that responsible for the suppression of immune responses and development of immune tolerance. Its critical role in the pathogenesis of various autoimmune diseases including systemic lupus erythematosus (SLE), rheumatoid arthritis (RA), and multiple sclerosis (MS) have been well-characterized (16). The possible link between dysregulation of Tregs and GD development was also indicated by several studies. When immunized with adenovirus expressing the TSHR A-subunit, mice lacking the CD4+CD25+ Tregs are more predisposed to develop GD compared to Tregs-intact mice (17). Moreover, patients with GD had significantly lower level of CD4+CD25+ Tregs than healthy controls, the lower level of CD4+CD25+ Tregs was associated with increased production of autoantibodies, which further suggested the pivotal role of Tregs in the loss of immune tolerance and development of autoimmune process in thyroid disorders (18).

**Figure 1:**
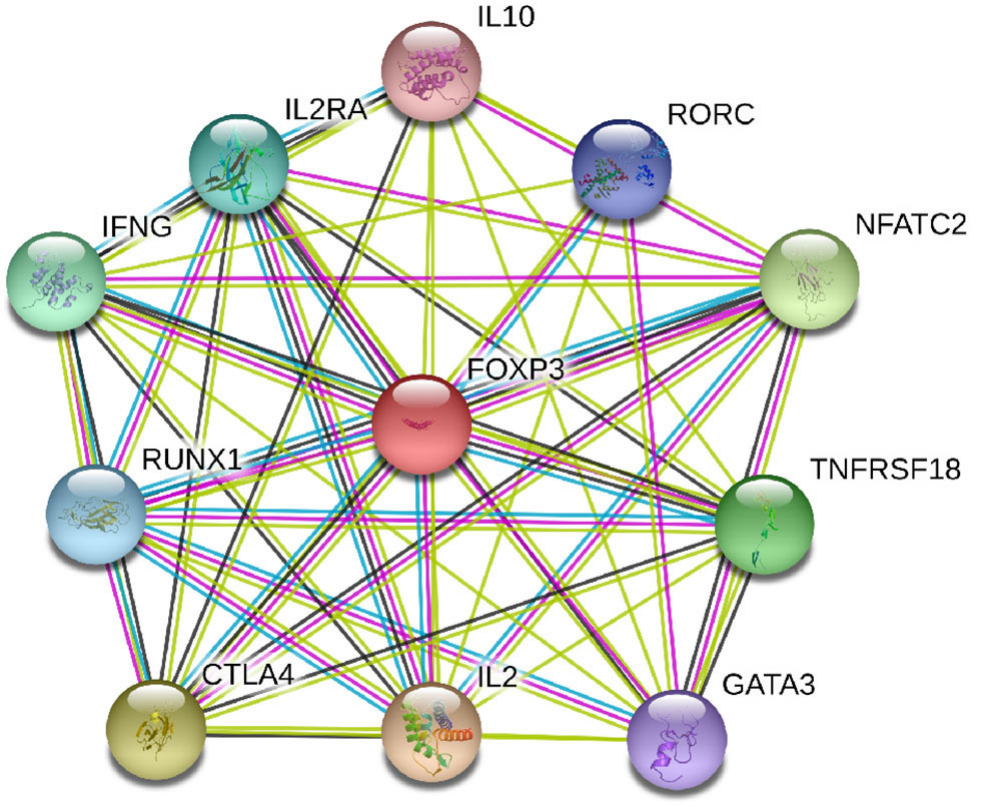
Protein-protein network of Foxp3 and its closest functional partners.

Foxp3 gene located on chromosome X is a major regulatory gene responsible for the differentiation of T cells into natural Tregs (19, 20). Through co-operation with nuclear factor of activated T cells (NFAT), which subsequently forms cooperative complexes with the AP-1 transcription factor family, Foxp3 controls the development and functioning of Tregs (21). Deficiency of Foxp3 gene caused by genetic variation impairs the suppressive function of Tregs and promotes autoimmunity (22). For instance, the rs3761548 allelic variation is capable of altering the Sp1 transcription factor binding efficiency which in turn affects Foxp3 gene expression (23). The protein-protein interaction (PPI) network of Foxp3 and its closest functional protein partners was shown in Figure 1. It illustrated that several proteins that closely interacted with Foxp3 were also involved in the pathogenesis of GD, such as IL-2, IL-2RA, IL-10, and CTLA4. Therefore, genetic variations in Foxp3 gene could be plausible candidate loci that contributed to the development of GD by means of changing the expression level of Foxp3. As a matter of fact, several association studies have been conducted to test this hypothesis but obtained inconsistent results. In these genetic association studies, rs3761547, rs3761548, and rs3761549 were the most commonly tested polymorphisms. Their genetic effect in the risk of GD has been investigated by a fair number of studies, particularly in the past 3 years. Since each individual study may lack the statistical power to test the modest genetic effect of each polymorphism due to inadequate sample size, we performed this meta-analysis based on the currently available evidence to quantitatively analyze the association between Foxp3 polymorphisms and risk GD.

## Methods

This systematic review and meta-analysis was carried out following the proposal for reporting Meta-analysis of Observational Studies in Epidemiology (MOOSE) guidelines (24).

### Literature Search Strategy

In order to identify observational studies that evaluated the association of Foxp3 polymorphisms and risk of GD, four online databases including PubMed, EMBASE, ISI Web of Science, and CNKI were searched up to December, 2019 without any language restriction. These databases were searched again before the submission of this manuscript in case that new articles were published in between. We combined medical subject headings (MeSHs) along with synonymous free texts to increase the sensitivity of the literature search. The search strings were slightly changed based on the specifications of each database. Here below was the search strategy for English databases: (“Polymorphism, Single Nucleotide”[Mesh] or Single Nucleotide Polymorphism or polymorphism or SNP or SNPs) and (“Graves Disease”[Mesh] or Grave Disease or Graves' Disease or Basedow's Disease or Basedow Disease or Exophthalmic Goiter or autoimmune thyroid disease or AITD) and (FOXP3 or transcription factor forkhead box P3 or Forkhead box protein 3). For CNKI, we used the search strategy as below: (Graves) and (Duo Tai Xing) and (Foxp3). The reference list of relative reviews and clinical guidelines was also manually checked for additional eligible studies.

### Inclusion and Exclusion Criteria

The inclusion and exclusion criteria were made according to the PICOS principle (participants, intervention, control, outcome, and study type): (i) participants in the GD group were patients with GD, the diagnosis should be made by according to well-established guidelines; (ii) the polymorphisms of interest included rs3761547, rs3761548, and rs3761549 in the Foxp3 gene; (iii) subjects in control group had no evidence of GD and were free of history of other autoimmune thyroid diseases; (iv) the primary outcome was the association between Foxp3 polymorphisms and risk of GD, as indicated by odds ratio (OR) and the associated 95% confidence interval (95%CI) which could be either provided by the original study or calculated based on the allele frequencies of the variant; (v) study type was required to be observational studies (case control study or cohort study). Case reports, case series, editorials, *in vitro* experiments, and animal studies were excluded. In case that multiple studies reported overlapping data, the most comprehensive one was selected by our study for a stronger statistical power. If only abstract was available, such as conference abstract, the corresponding author of the abstract would be contacted for raw data via email. The record would be abandoned if the author did not reply after we sent the email for at least three times.

### Risk of Bias Assessment

The Newcastle-Ottawa Scale (NOS) for the evaluation of methodological quality of non-randomized studies was recruited to assess the risk of bias in the included observational studies based on three broad perspectives: the selection of the study groups, the comparability of the groups, and the ascertainment of outcome of interest (25). Based on the scoring system ranging from 0 to 9, studies scoring 0 to 3 points, 4 to 6 points or 7 to 9 points were considered to have a high, a moderate or a low risk of bias, respectively. Two independent reviewers (LG and HL) performed the evaluation independently, the results were compared afterwards. The disagreement occurred during the evaluation process was resolved through discussion until a mutual consensus could be reached. Otherwise, the third reviewer (ZL) was consulted for opinions.

### Data Extraction

According to the predetermined eligibility criteria, two reviewers (HL and LG) finished the article screen process independently to collect qualified studies. Then data from these included studies were collected following a standardized data collection form which included name of the first author, year of publication, country of origin, ethnicity of participants, sample size, mean age, genotyping method, minor allele frequency in GD and control groups, Hardy-Weinberg Equilibrium (HWE) and main results.

### Quantitative Synthesis

The departure from HWE in control group was evaluated using the Chi-square test. To assess the potential relationship between Foxp3 polymorphisms and risk of GD, the estimated allelic effect (G vs. A for rs3761547, A vs. C for rs3761548, T vs. C for rs3761549) was expressed as OR and corresponding 95%CI using the genotype frequency data extrapolated from included studies. In our meta-analysis, we selected only the allelic model of inheritance to avoid an inflated type I error caused by multiple testing. Intra-study heterogeneity was estimated using a *Q*-test and the Higgins I-square test, where *P* > 0.1 and *I*^2^ < 50% indicated acceptable variability among included studies (26). Regardless of the magnitude of heterogeneity detected, we used the random-effect model to pool the data because of anticipated presence of between-study heterogeneity (27, 28). We assumed that the true genetic effect of Foxp3 polymorphisms was not the same among different populations.

Subgroup analysis by ethnicity was carried out to determine the different role of Foxp3 among different ethnicities. The leave-one-out sensitivity analysis was performed by removing each study at a time and reevaluating the resulting effect on the overall estimate. The forest plots were generated using RevMan 5.3 software (Copenhagen: The Nordic Cochrane Centre, The Cochrane Collaboration, 2014). Potential publication bias was detected using the Begg's rank correlation test and Egger's linear regression test by Stata version 12.0 (Stata Corp LP, USA), where *P* < 0.05 of either test indicated significant publication bias (29).

## Results

### Study Identification

**Figure 2:**
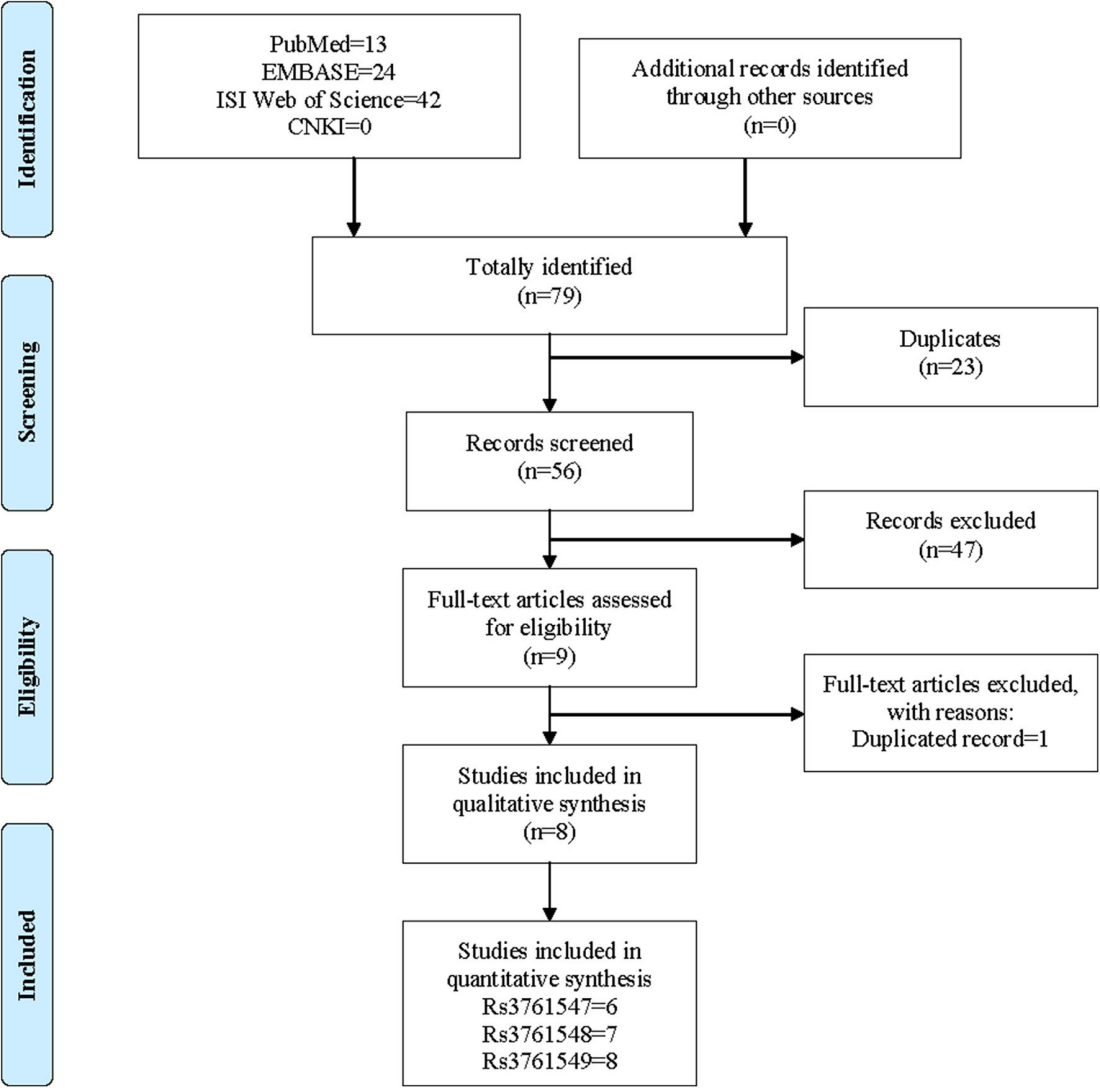
Flow chart of literature search and screen.

The initial literature search yielded a total of 79 records including 13 studies from PubMed, 24 studies from EMBASE, 42 studies from ISI Web of Science and 0 from CNKI. After the removal of 23 duplicated records, the remaining 56 studies were screened with titles and abstracts. At this stage, 46 studies were further excluded because of irrelevance, and 9 studies were downloaded for full-text screen. Finally, only 1 study was removed due to duplication, and 8 studies were deemed eligible to be included in the meta-analysis. The process of literature search and screen was presented in Figure 2.

### Main Characteristics and Quality Assessment

The main characteristics and findings of included studies were summarized in Table 1. Eight case control studies (30–37) involving 3,104 GD patients and 3,599 healthy controls were included in our meta-analysis. All the studies were published between 2006 and 2019, with the sample size ranging from 180 to 2,568. The majority of studies were performed in China (35–37) and India (31, 34), the remaining studies were carried out in Poland (30), UK (33), and Japan (32), respectively. Two widely used techniques for genotyping were used by our included studies, namely TaqMan and polymerase chain reaction-restricted fragment length polymorphisms (PCR-RFLP). Three different polymorphisms were investigated by the included studies, including six studies on rs3761547, seven studies on rs3761548, and eight studies on rs3761549. The main targeted population was early-middle adults, except for Bossowski's study (30) that focused on adolescents. All the studies reported baseline similarities in terms of age and gender.

**Table 1:** Main characteristics of included studies on the association between Foxp3 polymorphisms and risk of Graves' disease.

**References**	**Country**	**Ethnicity**	**Sample size (GD/HC)**	**Mean age (GD/HC)**	**Genotyping method**	**SNPs**	**MAF (GD/HC)**	**HWE**	**Associations**
Bossowski et al. (30)	Poland	Caucasian	145/161	16.5/16.3	TaqMan	rs3761547	14.11/8.19	<0.05	1.84 (1.02, 3.32)
						rs3761548	40.96/42.06	0.19	0.96 (0.67, 1.37)
						rs3761549	15.75/8.05	<0.05	2.13 (1.20, 3.80)
Fathima et al. (31)	India	Asian	80/285	33.9/32.0	PCR-RFLP	rs3761548	54.38/57.72	<0.05	0.87 (0.61, 1.24)
						rs3761549	41.25/33.51	0.28	1.39 (0.97, 2.00)
Inoue et al. (32)	Japan	Asian	109/71	32.7/44.1	PCR-RFLP	rs3761547	21.56/20.00	0.67	1.10 (0.65, 1.86)
						rs3761548	20.92/12.68	<0.05	1.82 (1.00, 3.33)
						rs3761549	21.84/23.39	0.52	0.92 (0.53, 1.59)
Owen et al. (33)	UK	Caucasian	633/528	NA	PCR-RFLP	rs3761549	12.69/14.32	NA	0.87 (0.67, 1.13)
Shehjar et al. (34)	India	Asian	135/150	38.0/NR	PCR-RFLP	rs3761547	21.85/20.33	0.15	1.10 (0.73, 1.64)
						rs3761548	40.00/31.00	0.08	1.48 (1.05, 2.10)
						rs3761549	35.93/18.00	0.35	2.55 (1.74, 3.76)
Yu et al. (35)	China	Asian	534/630	35.0/33.9	PCR-RFLP	rs3761547	25.00/22.94	<0.05	1.12 (0.93, 1.36)
						rs3761548	22.47/15.56	<0.05	1.57 (1.28, 1.94)
						rs3761549	27.06/21.9	0.29	1.32 (1.09, 1.60)
Yuan et al. (36)	China	Asian	1,100/1,468	36.0/45.0	TaqMan	rs3761547	NA	NA	1.03 (0.89, 1.19)
						rs3761548	NA	NA	1.03 (0.89, 1.20)
						rs3761549	NA	NA	1.02 (0.88, 1.18)
Zheng et al. (37)	China	Asian	368/ 306	39.7/41.6	PCR-RFLP	rs3761547	22.89/21.24	0.076	1.10 (0.84, 1.44)
						rs3761548	21.43/15.03	0.068	1.54 (1.15, 2.07)
						rs3761549	25.00/22.55	0.146	1.14 (0.88, 1.49)

*GD, Grave disease; SNP, single nucleotide polymorphism; MAF, minor allele frequency; HWE, Hardy-Weinberg Equilibrium; PCR-RFLP, polymerase chain reaction-restricted fragment length polymorphisms; NA, not applicable*.

The NOS was used for quality assessment of the included studies and the response of our included studies to each item of NOS was summarized in Table 2. The methodological quality of included studies was deemed to be moderate to high. All the included studies achieved at least 6 points with an average of 6.5 points.

**Table 2:** Quality assessment of included studies using the Newcastle-Ottawa Scale.

**References**	**Bossowski et al. (30)**	**Fathima et al. (31)**	**Inoue et al. (32)**	**Owen et al. (33)**	**Shehjar et al. (34)**	**Yu et al. (35)**	**Yuan et al. (36)**	**Zheng et al. (37)**
Adequate definition of cases	*	*	*	*	*	*	*	*
Representativeness of cases	*	–	–	–	–	–	–	–
Selection of control subjects	–	–	–	–	–	–	–	–
Definition of control subjects	*	*	*	*	*	*	*	*
Control for important factor or additional factor	**	*	*	*	**	*	**	*
Exposure assessment	*	*	*	*	*	*	*	*
Same method of ascertainment for all subjects	*	*	*	*	*	*	*	*
Non-response rate	*	*	*	*	*	*	*	*

*A study could be awarded a maximum of one star for each item except for the item “Control for important factor or additional factor”*.

The definition/explanation of each column of the Newcastle-Ottawa Scale is available from http://www.ohri.ca/programs/clinical_epidemiology/oxford.asp. *Suggested that cases and controls were matched for an important factor, **suggested that cases and controls were matched for at least an additional factor.

### Quantitative Analysis

#### Rs3761547

**Figure 3:**
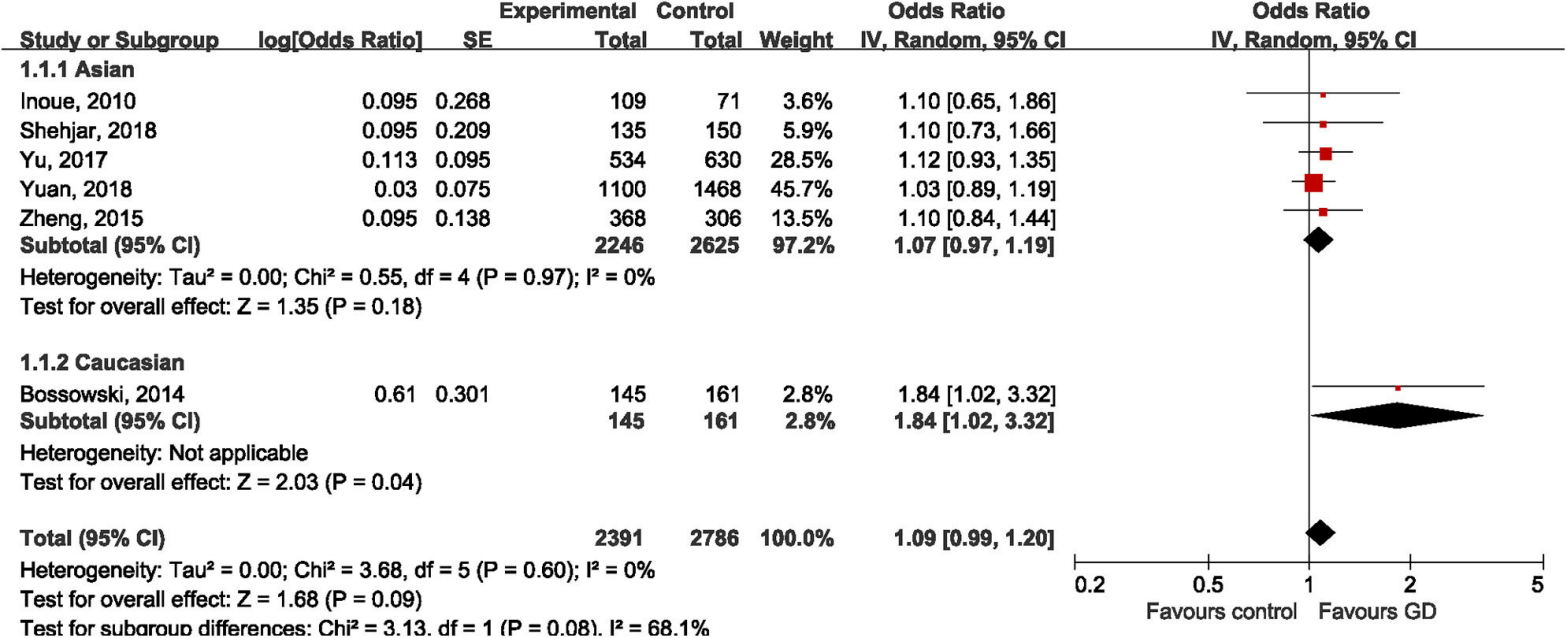
Forest plot of rs3761547 and risk of Graves' disease.

Six studies addressed the relationship between rs3761547 and risk of GD, including five studies (32, 34–37) on Asian population and one study (30) on Caucasian population. The pooled results showed that the minor allele of rs3761547 was not associated with risk of GD in the overall population (OR = 1.09, 95%CI 0.99, 1.20; *P* = 0.09), no significant heterogeneity was detected (*I*^2^ = 0%, *P* = 0.60). Subgroup analysis by ethnicity further confirmed the negative findings in Asians (OR = 1.07, 95%CI 0.97, 1.19; *P* = 0.18) but not among Caucasians (OR = 1.84, 95%CI 1.02, 3.32; *P* = 0.04), as shown in Figure 3. However, the genetic effect of rs3761547 among Caucasians cannot be confirmed since only one study on Caucasian was included. No significant publication bias was detected based on Begg's test (z = 0.38, *P* = 0.707) and Egger's test (*t* = 1.85, *P* = 0.137).

#### Rs3761548

**Figure 4:**
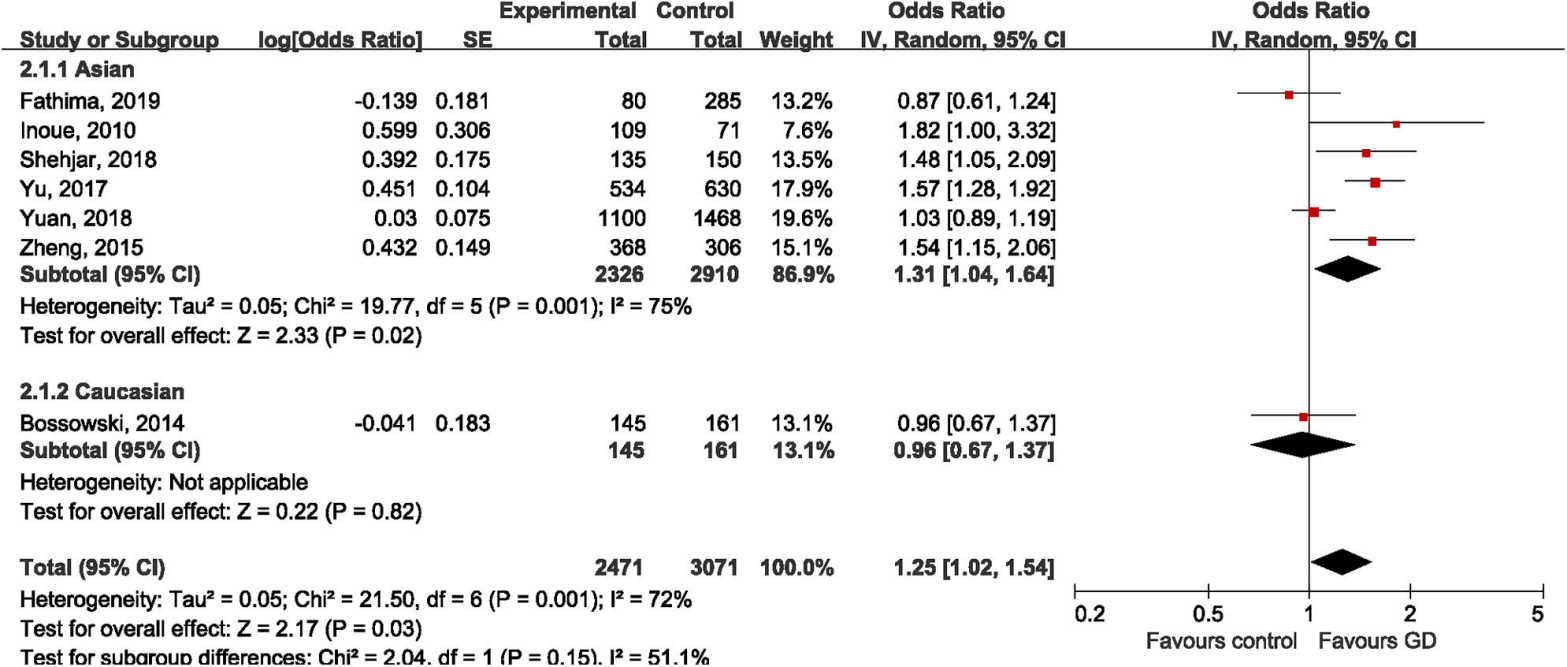
Forest plot of rs3761548 and risk of Graves' disease.

Seven studies (30–32, 34–37) investigated the genetic effect of rs3761548 on the risk of GD, however, achieving inconsistent results. Four studies (32, 34, 35, 37) reported significantly higher frequency of A allele in GD groups than control groups, while the other three studies (30, 31, 36) failed to replicate these positive findings. Under the random-effect model, the variant allele of rs3761548 polymorphism was associated with 25% higher risk of GD compared to reference allele in the overall population (OR = 1.25, 95%CI 1.02, 1.54; *P* = 0.03). Not surprisingly, significant heterogeneity was observed (*I*^2^ = 72%, *P* = 0.001). When stratified by ethnicity, we found a positive association among Asians (OR = 1.31, 95%CI 1.04, 1.64; *P* = 0.02) but not among Caucasians (OR = 0.96, 95%CI 0.67, 1.37; *P* = 0.82), as shown in Figure 4. The leave-one-out sensitivity analysis confirmed the statistical robustness of our findings (Table 3), and the study by Yuan et al. (36) was regarded as the major source of heterogeneity, since the intra-study heterogeneity decreased from 75 to 57% after the removal of Yuan's study. No publication bias was detected (Egger's test: *t* = 0.63, *P* = 0.554; Begg's test: z = 1.00, *P* = 1.00).

**Table 3:** Results of subgroup analysis by ethnicity and the leave-one-out sensitivity analysis.

**SNP**	**Subgroup**	**No. of studies**	**OR (95%CI)**	**P-OR**	**I^**2**^**	**P-het**	**Outlier study**	**Sensitivity analysis results**
rs3761547	Asian	5	1.07 (0.97, 1.19)	0.18	0%	0.97	NA	NA
	Caucasian	1	1.84 (1.02, 3.32)	0.04	NA	NA	NA	NA
	Overall	6	1.09 (0.99, 1.20)	0.09	0%	0.6	NA	NA
rs3761548	Asian	6	1.31 (1.04, 1.64)	0.02	75%	0.001	(36)	OR: 1.40 (1.12, 1.75), P-OR = 0.003, *I*^2^ = 57%, P-het = 0.06
	Caucasian	1	0.96 (0.67, 1.37)	0.82	NA	NA	NA	NA
	Overall	6	1.25 (1.02, 1.54)	0.03	72%	0.001	(36)	OR: 1.32 (1.05, 1.64), P-OR = 0.02, *I*^2^ = 63%, P-het = 0.02
rs3761548	Asian	6	1.30 (1.03, 1.64)	0.03	78%	0.0005	(34)	OR: 1.15 (1.01, 1.33), P-OR = 0.04, *I*^2^ = 35%, P-het = 0.19
	Caucasian	2	1.31 (0.55, 3.14)	0.54	87%	0.005	NA	NA
	Overall	8	1.28 (1.03, 1.58)	0.03	78%	<0.0001	(34)	OR: 1.15 (0.98, 1.36), P-OR = 0.10, *I*^2^ = 59%, P-het = 0.02

*SNP, single nucleotide polymorphism; OR, odds ratio; 95%CI, 95% confidence interval; NA, not applicable*.

#### Rs3761549

**Figure 5:**
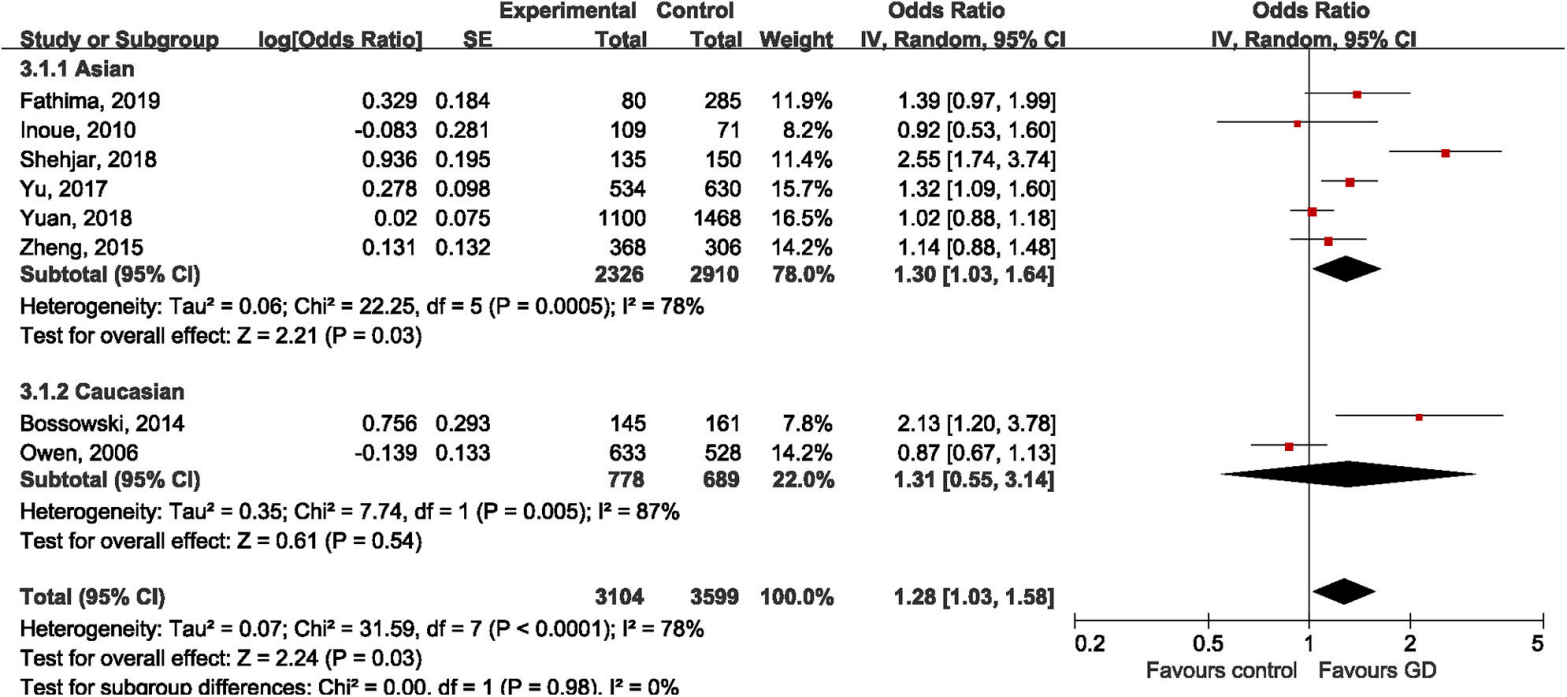
Forest plot of rs3761549 and risk of Graves' disease.

All the included studies tried to address the potential relationship between rs3761549 and risk of GD. The results of meta-analysis showed an increased risk of GD with the variant allele of rs3761549 in the overall population (OR: 1.28, 95%CI 1.03, 1.58; *P* = 0.03). Subgroup analysis by ethnicity showed different genetic effect of rs3761549 among Asians (OR: 1.30, 95%CI 1.03, 1.64; *P* = 0.03) and Caucasians (OR: 1.31, 95%CI 0.55, 3.14; *P* = 0.54) (Figure 5). The potential source of heterogeneity across studies was deemed to be Shehjar's study (34), as the intra-study heterogeneity decreased to 35% (*P* = 0.19) after its exclusion. No significant publication bias was observed (Egger's test: *t* = 1.34, *P* = 0.228; Begg's test: z=0.87, *P* = 0.386).

## Discussion

Foxp3 is predominantly expressed by CD4+CD25+ Tregs, it has been confirmed that normal Foxp3 expression is critical to maintain the suppressive function of Tregs. Therefore, genetic variations in Foxp3 gene causing deficient Foxp3 expression might contribute to the pathogenesis of GD by weakening the suppressive function of Tregs and thereby promote the autoimmune repsonse. Several genetic association studies have been conducted to test this hypothesis but the results remained controversial. Therefore, we for the first time collected and combined currently available evidence regarding the association between three common polymorphisms in Foxp3 and susceptibility of GD, to overcome the limitation of individual study, which is lack of power. The results of our meta-analysis supported no association of rs3761547 and risk of GD in Asians, the null association was further supported by sensitivity analysis and lack of intra-study heterogeneity. Evidence for rs3761547 and GD risk among Caucasians was still limited because only one study reported marginally increased risk of GD with the variant allele of rs3761547. The variant allele of both rs3761548 and rs3761549 was associated with increased risk of GD among Asians, possibly due to deficient Foxp3 expression and impaired function of Tregs, but neither polymorphism turned out to be related with GD among Caucasians. The negative results among Caucasians could be due to limited number of included studies, or as a result of ethnic difference. The variations are likely to have their own genetic ancestral background and if the alleles vary in frequency across populations, then different associations could be found. For instance, the MAF of rs3761549 in control subjects had an average frequency of 11.41% among Caucasians while it was 23.87% among Asians. The three common polymorphisms investigated by our study, rs3761547, rs3761548, and rs3761549 are all intronic variations and are not in linkage disequilibrium with other variants (38). Therefore, the association between these polymorphisms and risk of GD could be considered independent.

In terms of rs3761547, all the included studies among Asians consistently showed lack of association. The ORs from each individual study ranged from 1.03 to 1.12, yielding an overall estimate of 1.07 among Asians. To gain insight into the underlying mechanism, Yu et al., examined the correlation of Foxp3 polymorphisms and gene expression level using the RT-PCR. And it turned out that different genotypes of rs3761547 did not correlate with the expression level of Foxp3 in 220 subjects tested (35). In other words, variant allele of rs3761547 was not associated altered expression of Foxp3 and impaired function of Tregs. For Caucasians, only Bossowski et al. reported slightly increased frequency of C allele in patients with GD than healthy controls (*P* = 0.04) (30). This statistical difference then diminished down to a non-significant level when the analysis was restricted to females, which accounted for the majority of the enrolled subjects. Therefore, we cannot make conclusion regarding the association between rs3761547 and risk of GD among Caucasians. Furthermore, the findings among Caucasian population should be interpreted with caution due to the inadequate samples size (*n* = 306) and departure of HWE in the controls (*P* < 0.05).

The molecular mechanisms by which rs3761548 polymorphisms affects Foxp3 expression has been well-characterized in psoriasis (39). Based on a large-scale SNP analysis, the AA genotype of rs3761548 was able to cause loss of bindings to the E47 and c-Myb factors, leading to defective transcription of Foxp3 gene (39). Inoue et al. found that 11.3% of the patients with intractable GD had the AA genotype with the lowest production of Foxp3 among three different genotypes of rs3761548 (CC, CA, and AA), while AA was absent in patients with GD in remission (32). It was speculated that AA genotype of this polymorphism contributed to the severity of autoimmune response. This hypothesis was supported by a recent study, in which Yu et al. reported the negative correlation between the presence of each A allele and decreased expression level of Foxp3 gene (35). Another *in vitro* study also indicated that the luciferase activity of Foxp3 promoter was reduced by more than 40% with the mutation from C to A at the rs3761548 locus (37). According to the results of our meta-analysis, a significant allelic effect of the variant allele was observed in GD patients, which was indeed suggestive of the genetic effect of rs3761548 in conferring an increasing predisposition to GD. Four out of seven included studies (32, 34, 35, 37) reported significantly higher frequency of A allele in GD patients than controls, with an estimate of OR ranging from 1.48 to 1.82, whereas the other three studies (30, 31, 36) included negative findings. The main source of heterogeneity among Asians was deemed to be Yuan's study, since the intra-heterogeneity decreased from 75 to 57% after its removal (36). The results of Yuan's study might be biased because the healthy controls were significantly older than patients with GD. Besides, Yuan et al. did not report allele frequency, genotype distribution as well as results of HWE, which hampered further evaluation of this study. Nevertheless, the drawn conclusion remained significant even after the exclusion of Yuan's study, suggesting the reliability of our data synthesis. Again, the evidence among Caucasian population was still limited as only one study was conducted within Caucasian background (30).

For rs3761549, our results indicated that the variant allele was associated with 28% higher risk of GD compared with the reference allele (OR: 1.28, *P* = 0.03). Similar as rs3761548, rs3761549 might confer an increased susceptibility of GD through disrupting the normal transcriptional activity of Foxp3 and thereby leading to impaired Tregs function and enhanced autoimmune response. Yu et al. found that Foxp3 expression level was negatively affected by the T allele (variant allele) and the highest level of Foxp3 expression was detected among CC carriers, decreasingly followed by CT and TT carriers (35). The biological importance of rs3761549 was also reported by Zheng's study, in which TT carriers had significantly higher level of FT3 than CT and/or CC carriers (37). It would be of great interest to determine the level of autoantibodies in GD patients carrying different genotypes. If TT or CT genotypes are associated with increased level of autoantibodies, or if they are more frequent in intractable GD than GD with remission, the genotype information might serve as a novel therapeutic target and facilitate early and efficient diagnosis. Despite the significant association observed in Asians, the source of heterogeneity should be identified before we accepted the estimate of effect. The sensitivity analysis suggested that study by Shehjar et al. could be the major source of variability due to limited sample size (*n* = 285), and the heterogeneity decreased from 78 to 35% after the omission of this study (34). The robustness of our conclusion was also confirmed by the sensitivity analysis, since the significant association remained unchanged after the omission of Shehjar's study.

Several strengths and limitations should not be ignored when interpreting the results of our study. Strengths included: (i) all tissues sources were blood, and the genotype was confirmed using well-established method; (ii) given that the majority of included studies lacked the power to detect modest effect of Foxp3 polymorphisms (*n* < 1,000), we provided a precise estimation of the effect by increasing statistical power with a total of 6,703 subjects included; (iii) the methodological quality of included studies was regarded as moderate to high based on the NOS scoring system; (iv) publication bias was not detected in any of the combination; (v) we selected only the allele model of inheritance for data combination to avoid an inflated type I error; (vi) the sensitivity analysis successfully identified potential source of heterogeneity, more importantly, the omission of these outliers did not change the overall estimate of our meta-analysis.

Several limitations in our study should also be acknowledged: (i) the sample size of each individual study was not adequate enough to detect the modest genetic effect of Foxp3 polymorphisms on GD risk; (ii) all the included studies were hospital-based, not population-based, so the findings could not be directly generalized to the general population; (iii) the strength of associations was indicated as crude OR and 95%CI, which was calculated using the genotype frequency or provided by the original study. The confounding due to gene-gene and gene-environment interaction cannot be ruled out; (iv) we failed to stratify the studies by sex or clinical variables such as TPOAb, TgAb, and TRAb because the included studies did not provide sufficient data. For complex and clinically heterogeneous diseases like autoimmune thyroid disease, even clinically similar patients may represent different subtypes that cannot be distinguished using currently available clinical manifestations or laboratory parameters. If a genetic variant is only present in some specific subtypes, such as intractable GD, its genetic effect in intractable GD could be underestimated when intractable GD and GD in remission are included in the same study. Similarly, future studies should also distinguish GD with and without Graves' ophthalmopathy (GO); (v) the majority of our concluded studies were conducted in Asian population, only two studies investigated the genetic effect of Foxp3 polymorphisms in Caucasian population. Although no publication bias was detected for all three meta-analyses, the tests remained underpowered as no more than ten studies were included. More studies within different ethnic backgrounds are warranted.

## Conclusion

Taken together, the variant allele of rs3761548 and rs3761549 in Foxp3 gene was associated with increased of GD among Asians, possibly because both polymorphisms negatively affect the transcriptional activity of Foxp3 and lead to impaired function of Tregs. The genetic effect of Foxp3 polymorphisms in Caucasians cannot be determined as only limited number of studies has been conducted so far. It will be of great interest to dive deeper into the possible link of certain Foxp3 polymorphisms and thyroid-specific antibodies, to facilitate our understanding of the immune regulation at a molecular level as well as Tregs-related pathogenesis that occur during autoimmune thyroid diseases. Future large-scale and well-designed studies are still needed to confirm our findings, particularly in different ethnic background.

## Data Availability Statement

The raw data supporting the conclusions of this article will be made available by the authors, without undue reservation.

## Author Contributions

ZL and YD put forward the conception and design of this study. HL and XL collected the data performed electronic searches for relevant studies in this meta-analysis. HL and ZL wrote the manuscript. ZY made the critical revision of the manuscript. All authors read and approved the final manuscript.

## Conflict of Interest

The authors declare that the research was conducted in the absence of any commercial or financial relationships that could be construed as a potential conflict of interest.
